# Supplementary material for: Empirical methods for controlling false positives and estimating confidence in ChIP-Seq peaks
Source: BMC Bioinformatics. 2008 Dec 5;9:523. doi: 10.1186/1471-2105-9-523 (PMC2628906; doi:10.1186/1471-2105-9-523)
Supplement: Additional File 1 — A variety of html documents from the USeq web site detailing the available applications, their best usage, output file type descriptions, command line menus, etc. [file 1471-2105-9-523-S1.zip › USeqUserGuides/JavaDoc.zip!/JavaDoc/util/bio/annotation/class-use/GeneRep.html]

Uses of Class util.bio.annotation.GeneRep


|  |  |  |
| --- | --- | --- |
| **Overview** \| **Package** \| **Class** \| **Use** \| **Tree** \| **Deprecated** \| **Index** \| **Help** | |  |
| PREV   NEXT | **FRAMES**    **NO FRAMES**     **All Classes** |  |


---


## **Uses of Class util.bio.annotation.GeneRep**

| Packages that use GeneRep | |
| --- | --- |
| **gata.geneGlyphs** | For representing annotation, scale bars, and generic tracks. |
| **util.bio.annotation** | For representing gene annotation. |

Uses of GeneRep in gata.geneGlyphs

Constructors in gata.geneGlyphs with parameters of type GeneRep

`GeneRepGlyph(GeneRep geneRep,
GATAParams gataParams,
AnnoSpecParams annoParams)`

Uses of GeneRep in util.bio.annotation

| Methods in util.bio.annotation that return GeneRep | |
| --- | --- |
| `GeneRep` | `GeneGroup.getGeneRep()` |

---


|  |  |  |
| --- | --- | --- |
| **Overview** \| **Package** \| **Class** \| **Use** \| **Tree** \| **Deprecated** \| **Index** \| **Help** | |  |
| PREV   NEXT | **FRAMES**    **NO FRAMES**     **All Classes** |  |


---
